# Effectiveness of tumor necrosis factor inhibitors in children with enthesitis-related arthritis: a single-center retrospective analysis

**DOI:** 10.3389/fped.2023.1122233

**Published:** 2023-05-26

**Authors:** Tonghao Zhang, Shuoyin Huang, Yanli Guo, Jing Jin, Wu Yan, Panpan Wang, Yuying Fang, Yingying Liu, Yuting Pan, Zhidan Fan, Haiguo Yu

**Affiliations:** ^1^ Department of Rheumatology and Immunology, Children’s Hospital of Nanjing Medical University, Nanjing, China; ^2^ Department of Child Health Care, Children’s Hospital of Nanjing Medical University, Nanjing, China

**Keywords:** juvenile idiopathic arthritis, enthesitis-related arthritis, anti-tumor necrosis factor inhibitor, laboratory parameters, treatment, outcomes

## Abstract

**Objective:**

In children with enthesitis-related arthritis (ERA), the hip and sacroiliac joint function might be impaired if not properly treated. We sought to evaluate the effectiveness of anti-tumor necrosis factor-α (TNF-α) therapy using the inflammatory indicators, Juvenile Arthritis Disease Activity Score 27 (JADAS27) and magnetic resonance imaging (MRI).

**Methods:**

We conducted a single-center retrospective study of 134 patients with ERA. We evaluated the effect of anti-TNF therapy on the inflammatory indicators, active joint count, MRI quantitative score, and JADAS27 over 18 months. We used the Spondyloarthritis Research Consortium of Canada (SPARCC) and the Hip Inflammation MRI Scoring System (HIMRISS) scoring systems for hip and sacroiliac joints scoring.

**Results:**

The average age of onset of children with ERA was 11.62 ± 1.95 years, and they were treated with disease-modifying antirheumatic drugs (DMARDs) combined with biologics (*n* = 87, 64.93%). There were no differences in HLA-B27 positivity between the biologics and non-biologics treatment groups [66 (49.25%) vs*.* 68 (50.75%), *P* > 0.05]. Children who received anti-TNF (71 received etanercept, 13 adalimumab, 2 golimumab, and 1 infliximab) therapy improved significantly. Children with ERA used DMARDs and biologics at baseline (Group A) were followed up to 18 months, and their active joint count (4.29 ± 1.99 vs. 0.76 ± 1.33, *P* = 0.000), JADAS27 (13.70 ± 4.80 vs. 4.53 ± 4.52, *P* = 0.000) and MRI quantitative scores (*P* = 0.001) were significantly lower than those at baseline. Some of the patients (*n* = 13, 9.70%) were treated with DMARDs at the onset of the disease, but did not show significant improvement (Group B). After 6−18 months of switching to anti-TNF therapy, related indicators of the children were significantly lower than at baseline and 1 month (*P* < 0.013). At 18 months, a total of 33 patients (*n* = 74, 44.59%) in Group A and 7 (*n* = 13, 53.85%) in Group B reached inactive state.

**Conclusion:**

Eighteen months after diagnosis, anti-TNF therapy was found to be effective in children diagnosed with ERA. MRI is important for the early diagnosis of juvenile idiopathic arthritis. TNF-α inhibitors can significantly improve the clinical manifestations of sacroiliac joint and hip involvement in patients with ERA. Overall, the real-world study provides more evidence for precision diagnosis and treatment for other hospitals, families and patients.

## 1. Introduction

Juvenile idiopathic arthritis (JIA) is a form of inflammatory arthritis affecting children. Enthesitis-related arthritis (ERA) is a form of juvenile idiopathic arthritis (JIA), defined by the International League of Associations for Rheumatology (ILAR) (1) to primarily affect the entheses and peripheral joints, but also involve the axial skeleton. The clinical manifestations of ERA are chronic progressive arthritis, asymmetric impairment of the lower limb and sacroiliac joints, acute anterior uveitis, low back pain, negative serum rheumatoid factor (RF), positive HLA-B27, and familial aggregation tendency. ERA is common in children >6 years. If not diagnosed and treated early, it can cause joint destruction and disability with poor prognosis, and most patients eventually developing ankylosing spondylitis. Disease activity and structural changes can adversely affect patients' long-term physical function and quality of life (2–6). The Pediatric Rheumatology International Trials Organization (PRINTO) classified JIA anew in 2018 using four disorders: (a) systemic JIA, (b) RF-positive JIA, (c) enthesitis/spondylitis-related JIA, (d) early onset antinuclear antibody-positive JIA, and (e) other forms (to be analyzed in future studies) (7). Treatment with anti-tumor necrosis factor (TNF)-α antibody effectively can reduce active inflammatory lesions by up to 80% in 6–24 months, as shown by magnetic resonance imaging (MRI) (8). This reduction would also be expected to halt the progression of ankylosing spondylitis; however, no significant reduction in spine ossification and fusion has been reported and any reduction was not as significant as that of non-steroidal anti-inflammatory drugs (NSAIDs) (9, 10). Therefore, our study aimed to thoroughly investigate the effectiveness of anti-TNF from the onset of illness to 18 months in children with ERA using laboratory tests, MRI quantitative scoring, and the Juvenile Arthritis Disease Activity Score 27 (JADAS27).

## 2. Materials and methods

This study is a single-center clinical retrospective analysis and involving 134 children diagnosed with ERA from January 2018 to March 2021 in our institute, and who met the newly revised JIA classification and the ILAR diagnostic criteria from 2004. ERA is closely associated with IBD and/or subclinical gut inflammation. According to the norms, calprotectin tests was prescribed for all the ERA patients and gastroscopy and colonoscopy examination were subsequently conducted for those with high faecal calprotectin. IBD and/or subclinical gut inflammation were excluded from our study. The long-term treatment and follow-up of all patients were conducted by rheumatologists. All children with ERA were followed up five times for ≥18 months and all children with ERA underwent MRI examination at baseline, 1, 6, 12 and 18 months as the inclusion criteria. Demographics, clinical characteristics, laboratory data, treatment effect, and MRI quantitative scores data from onset to 1, 6, 12, and 18 months were collected and analyzed. Patients of 6–17 years who fulfilled the ILAR criteria rather than the new PRINTO standard were deemed eligible. As demonstrated by clinical data, ERA often involves both the sacroiliac and hip joints at the time of diagnosis. The inclusion criteria for sacroiliac joint involvement were: (i) occult pain in the lower back, buttocks, proximal thighs, and groin and (ii) morning stiffness, albeit usually lasting <30 min. The clinical manifestations of hip involvement were (i) spontaneous groin, thigh, and hip pain with or without a history of trauma; (ii) limited internal and external rotation of the symptomatic hip(s) after the initial clinical assessment; and (iii) both acute and chronic inflammatory changes on MRI (11).

Demographic profiles of newly diagnosed children with ERA, individual and family history of inflammatory and autoimmune diseases or psoriasis, time from symptom onset to diagnosis, site of joints involved, active and restrictive joint count, acute uveitis, joint effusion, medication, and related laboratory tests, such as C-reactive protein (CRP), erythrocyte sedimentation rate (ESR), HLA-B27, platelet count, alanine aminotransferase, and aspartate aminotransferase, were recorded and analyzed. Serum CRP level and ESR were measured at baseline and at 1, 6, 12, and 18 months. Joint lesions can show bone marrow edema (BME), bone erosion, and joint effusion on MRI. During follow-up, each patient's medications, such as NSAIDs (e.g., diclofenac sodium, naproxen, elecoxib), disease-modifying antirheumatic drugs (DMARDs) (e.g., methotrexate, sulfasalazine), and TNF-α blockers (adalimumab, etanercept, infliximab) were recorded. At the same time, rheumatologists can comprehensively evaluate the disease activity in children by assessing the CRP, ESR, MRI quantitative score, active joint count, physician and parent visual analog scale score, and JADAS27. Acute-phase reactants are objective measures of inflammatory response. Among them, ESR is more sensitive for the detection of disease activity, while other inflammatory response indicators including CRP, white blood cell, and platelet counts are not sensitive (12). At the same time, studies have shown that in the absence of laboratory indicators, JADAS27 can still assess disease activity (13).

Quantitative assessment of the treatment changes in bone marrow edema of children's sacroiliac and hip joints by MRI, often use the Spondyloarthritis Research Consortium of Canada (SPARCC) and the Hip Inflammation MRI Scoring System (HIMRISS).

During the study, children with ERA who received biological agents from baseline to the end of the follow-up were included in Group A, while those who were initially treated with non-biological agents but did not show significant improvement were included in Group B.

This study was approved by the Ethics Committee of Children's Hospital of Nanjing Medical University (202008041-1).

### 2.1. SPARCC scoring system

This scoring method is based entirely on the assessment of an increased signal on T2 with fat suppression or short tau inversion recovery (STIR) sequences denoting bone marrow edema on oblique coronal slices of the SI joint. All signal changes within the iliac bone and sacrum up to the sacral foramina were scored on six consecutive slices through the SI joint. These slices were selected based on the SPARCC protocol, which defines the acquisition characteristics and encompasses most of the synovial compartment. The sacral interforaminal bone marrow STIR signal forms a reference for the determination of increased signals in the SI joints. Each SI joint was divided into four quadrants, and the presence of an increased STIR signal in each of these four quadrants was recorded in each of the six slices, giving a maximum score of 48. The presence of a lesion exhibiting either an intense signal (comparable to the signal from adjacent blood vessels) or depth of ≥1 cm anywhere within each SI joint of the six slices was given an additional score (i.e., a maximum additional score of 4 per slice), bringing the total score to 72 (14).

### 2.2. HIMRISS scoring system

The HIMRISS was used to assess the changes on MRI from baseline to 18 months. MRI was performed with a coronal STIR sequence with a slice thickness of 4 mm and a field of view of 400  ×  400 mm. The baseline and follow-up MRI examinations of both hips had to be performed every 6 months during the 18-month follow-up period. The MRI scans at baseline and at 18 months were calculated using the HIMRISS. The HIMRISS protocol was used to score the inflammatory changes on the MRI of the hip joints, including the BME of the femoral head and acetabulum, and synovitis (15, 16).

### 2.3. Statistical analyses

This retrospective, self-controlled study adopted a single-sample design. The data in this study were verified to conform to a normal distribution. A Paired T-test was used to assess the differences in remission of clinical indicators (CRP, ESR, active joint count, and JADAS27) and MRI improvement between different follow-up time points (baseline and 1, 6, 12, and 18 months), and use Bonferroni test correction, which makes the data more accurate and meaningful. Measurement data are expressed as mean ± standard deviation (x ± s), and enumeration data as frequency and percentage. Statistical analyses were performed using IBM SPSS 23.0 (IBM Corp., Armonk, NY, USA). Statistical significance was set to *P* < 0.013.

## 3. Results

### 3.1. Demographic and clinical characteristics

We recruited 134 children with ERA from January 2018 to March 2021, and treatment follow-up was carried out for 18 months. The baseline demographic characteristics of the children diagnosed with ERA are shown in Table 1. Among them, 121 (90.3%) were male and the ratio of boys to girls was 9.31:1. The x ± s age at diagnosis was 11.62 ± 1.95 years. The time from symptom onset to diagnosis was 10.48 ± 8.24 months. Of the 134 children, 66 (49.25%) were HLA-B27 positive and 31 (23.13%) had a family history of HLA-B27-related diseases in first-degree relatives. Besides, in Group A (*n* = 74), 43 patients (58.11%) with ERA were HLA-B27 positive. In Group B (*n* = 13), 6 (46.15%) children with ERA were HLA-B27 positive, with no significant difference (*P* > 0.05). There were only five cases of acute uveitis and 15 cases of inflammatory back pain. In addition, of the children with joint pain, 104 had one or more joint effusions.

**Table 1:** Baseline patients with enthesitis and disease characteristics (*n* = 134).

Characteristic	*N*
**Demographics**	
Age at diagnosis, mean ± SD years	11.62 ± 1.95
Time between onset of symptoms and diagnosis, mean ± SD months	10.48 ± 8.24
Male sex, *n* (%)	121 (90.30)
HLA-B27 presence, *n* (%)	66 (49.25)
A family history positive for HLA-B27 related diseases, *n* (%)	31 (23.13)
Uveitis, *n* (%)	5 (3.73)
**Clinical features and patient-reported outcomes at diagnosis, median (IQR)**	
Active joint count	4 (1–14)
Restricted joint count	1 (0–7)
JADAS27	12.95 (3–34)
MRI score (Sacroiliac)	10 (0–32)
MRI score (Hip)	4 (0–9)
Patient/parent disease activity (VAS 0–10)	5 (2–9)
Physician disease activity (VAS 0–10)	4 (1–8)

### 3.2. Laboratory tests of children with ERA

During the 18-month follow-up period, all enrolled patients underwent clinical symptom assessment and MRI examination. Laboratory tests, such as CRP (19.57 ± 20.33 vs. 7.34 ± 2.84, *P* = 0.000), and ESR (29.66 ± 25.51 vs. 10.30 ± 16.97, *P* = 0.000) in Group A, were significantly lower between each visit and the baseline (Table 2, *P* < 0.013). However, in Group B, at one month of follow-up, ESR (24.38 ± 22.10 vs. 36.54 ± 23.75, *P* = 0.052) and CRP (19.23 ± 19.75 vs. 25.15 ± 22.43, *P* = 0.125) did not improve significantly or worsened compared with baseline. Subsequently, the treatment of ERA children in Group B was converted to anti-TNF, and at 18 months of follow-up, CRP (7.00 ± 1.84, *P* = 0.000) and ESR (8.54 ± 5.46, *P* = 0.010) were significantly lower than that at baseline. There were no significant differences in leukocytes, platelets, alanine aminotransferase, and aspartate aminotransferase during follow-up (*P*>0.013). Laboratory measures declined with the treatment. This indicates that biological agents can improve the inflammatory indicators of children with ERA.

**Table 2:** Laboratory tests at the onset of ERA (Group A and Group B).

Laboratory test	Baseline	1 Month	*P*	6 Months	*P*	12 Months	*P*	18 Months	*P*
CRP (0–10)	19.57 ± 20.33	13.59 ± 14.99	0.007	11.27 ± 13.09	0.000	9.59 ± 9.29	0.000	7.34 ± 2.84	0.000
ESR (≤20)	29.66 ± 25.51	21.49 ± 21.69	0.006	18.00 ± 19.43	0.000	13.62 ± 16.76	0.000	10.30 ± 16.97	0.000
WBC (4–10)	8.11 ± 1.97	7.95 ± 2.38	0.153	7.45 ± 2.03	0.007	7.27 ± 1.90	0.001	6.89 ± 1.40	0.000
PLT (100–300)	351.38 ± 72.23	339.83 ± 74.85	0.076	318.76 ± 77.01	0.001	303.03 ± 66.23	0.000	279.35 ± 72.99	0.000
ALT (7–30)	13.26 ± 9.10	32.56 ± 146.11	0.056	15.62 ± 14.08	0.049	14.45 ± 9.92	0.104	15.51 ± 15.16	0.106
AST (14–44)	19.00 ± 6.52	34.70 ± 119.08	0.056	20.38 ± 8.79	0.061	20.02 ± 7.17	0.082	22.16 ± 8.81	0.041
**Switch to anti-TNF**									
CRP (0–10)	19.23 ± 19.75	25.15 ± 22.43	0.125	7.08 ± 2.16	0.011	7.42 ± 2.00	0.000	7.00 ± 1.84	0.000
ESR (≤20)	24.38 ± 22.10	36.54 ± 23.75	0.052	10.15 ± 6.58	0.011	9.46 ± 4.68	0.006	8.54 ± 5.46	0.010
WBC (4–10)	7.62 ± 1.15	7.15 ± 2.06	0.125	7.33 ± 1.46	0.149	7.51 ± 2.33	0.223	7.61 ± 1.40	0.248
PLT (100–300)	323.92 ± 74.08	331.92 ± 73.54	0.198	318.54 ± 73.06	0.215	316.00 ± 60.50	0.194	295.23 ± 74.53	0.089
ALT (7–30)	14.23 ± 6.31	19.69 ± 14.08	0.058	19.69 ± 16.41	0.073	17.54 ± 8.71	0.074	16.54 ± 10.29	0.129
AST (14–44)	19.77 ± 4.47	23.15 ± 5.52	0.028	22.31 ± 6.22	0.066	21.77 ± 6.61	0.099	23.31 ± 6.91	0.037

(CRP, C-reaction protein; ESR, erythrocyte sedimentation rate; WBC, white blood cell; PLT, platelet count; ALT, alanine aminotransferase; AST, aspartate aminotransferase, *P*-values are corrected by Bonferroni test and *P *< 0.013 is considered meaningful).

### 3.3. Quantitative MRI assessment

**Figure 1:**
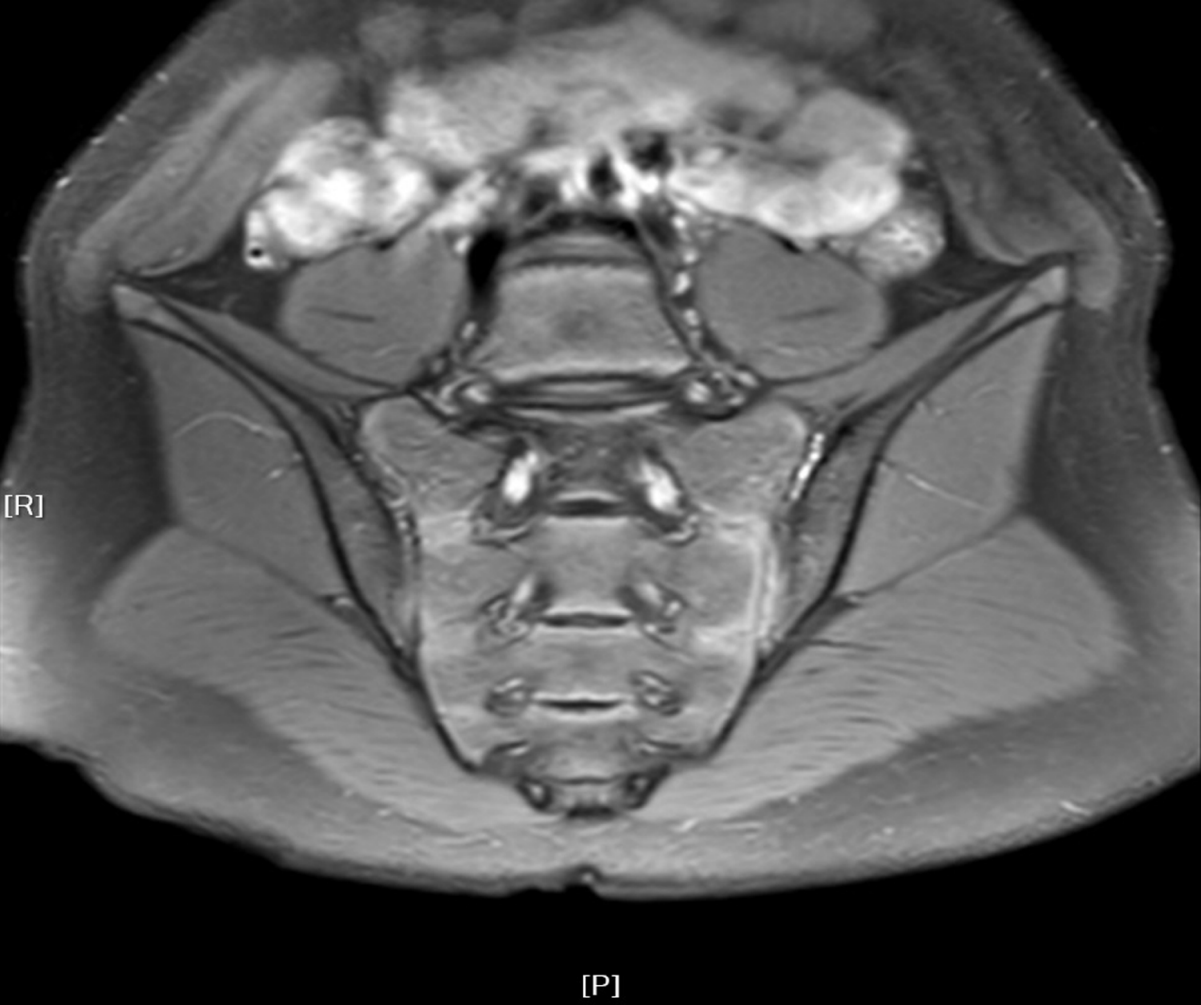
Bone marrow edema of sacroiliac joints.

**Figure 2:**
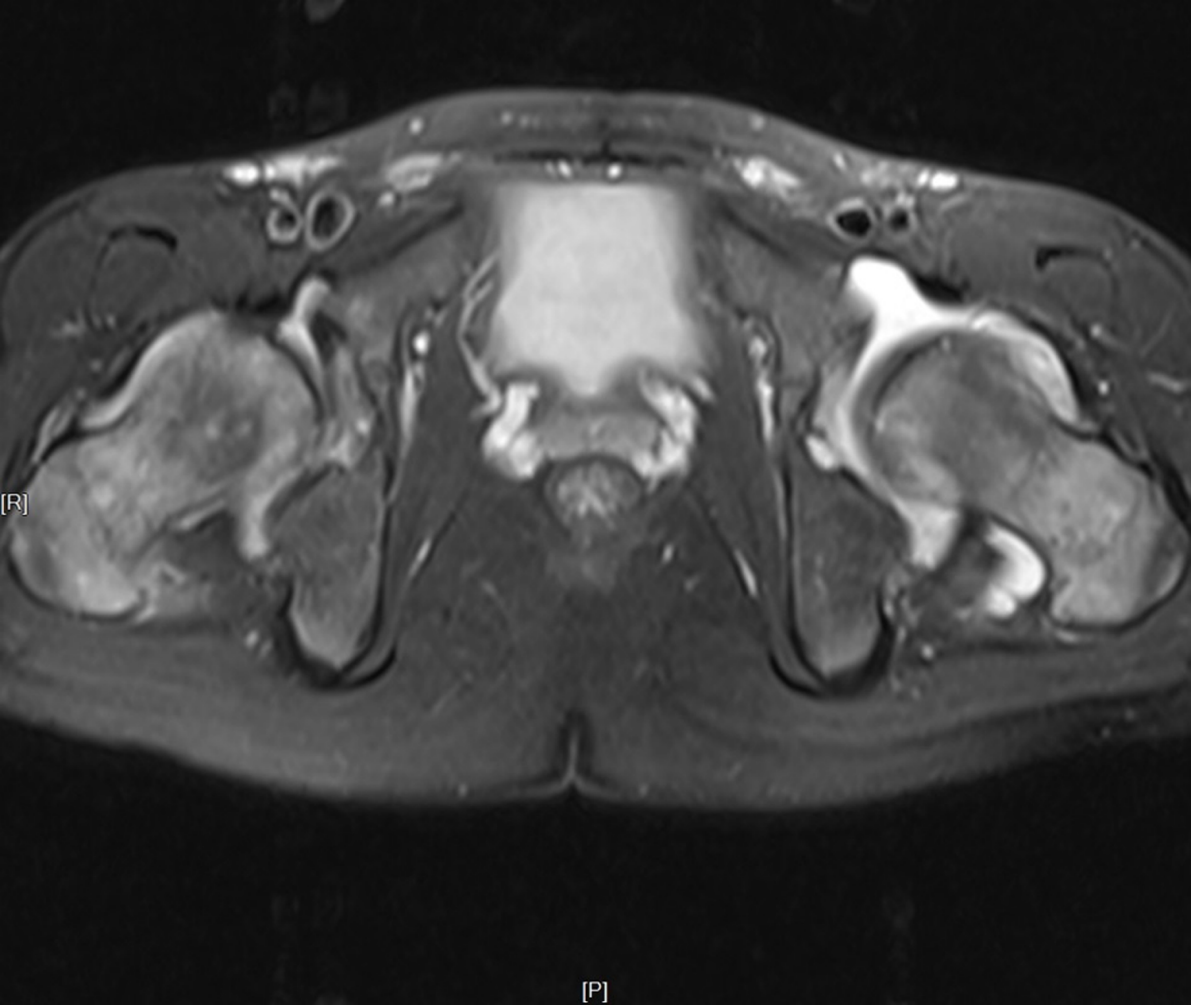
Bone marrow edema of hip joints.

Children with ERA in Groups A and B received an MRI at each follow-up visit (Figures 1, 2). In group A, the quantitative scores of sacroiliac joints (10.68 ± 6.96) and hip joint (3.61 ± 2.27) were relatively high at baseline, and with the progress of anti-TNF treatment, the quantitative scores of sacroiliac joints (1.93 ± 2.29, *P* = 0.000) and hip (0.48 ± 0.81, *P* = 0.001) decreased significantly compared with baseline. In Group B, the children were transferred to biological agents due to the poor treatment effect of non-biological agents in January. As treatment progresses, the MRI quantitative scores of sacroiliac joints (1.92 ± 2.37) and hip joint (0.62 ± 1.21) were significantly lower than at baseline (sacroiliac joints 9.69 ± 5.28, hip joint 4.08 ± 2.43, *P* = 0.000) and 1 month (sacroiliac joints 9.15 ± 5.63, hip joint 4.08 ± 2.23, *P* = 0.000) (Table 3). Therefore, MRI quantitative scoring is of great significance for detecting and evaluating of early bone marrow edema in children with ERA.

**Table 3:** Related indicators for the evaluation of disease activity (Group A and Group B).

	Baseline	1 Month	*P*	6 Months	*P*	12 Months	*P*	18 Months	*P*
MRI score (Sacroiliac)	10.68 ± 6.96	7.95 ± 5.26	0.001	5.80 ± 4.35	0.001	3.86 ± 3.42	0.000	1.93 ± 2.29	0.000
MRI score (Hip)	3.61 ± 2.27	2.524 ± 1.81	0.000	1.71 ± 1.55	0.001	1.02 ± 1.17	0.000	0.48 ± 0.81	0.001
JADAS27	13.70 ± 4.80	10.13 ± 5.55	0.000	8.69 ± 4.28	0.000	7.08 ± 4.08	0.000	4.53 ± 4.52	0.000
Active joint count	4.29 ± 1.99	1.98 ± 2.24	0.000	1.46 ± 1.80	0.000	1.03 ± 1.42	0.000	0.76 ± 1.33	0.000
Patient/parent disease activity (VAS 0–10)	4.84 ± 1.07	4.48 ± 1.32	0.013	4.63 ± 0.77	0.037	4.28 ± 1.59	0.002	2.79 ± 2.07	0.000
Physician disease activity (VAS 0–10)	4.44 ± 1.55	3.68 ± 1.66	0.001	2.95 ± 1.37	0.000	2.68 ± 1.08	0.000	2.18 ± 1.47	0.000
**Switch to anti-TNF**									
MRI score (Sacroiliac)	9.69 ± 5.28	9.15 ± 5.63	0.203	5.31 ± 2.84	0.005	3.23 ± 2.33	0.000	1.92 ± 2.37	0.000
MRI score (Hip)	4.08 ± 2.43	4.08 ± 2.23	0.234	2.08 ± 1.86	0.008	0.69 ± 0.72	0.000	0.62 ± 1.21	0.000
JADAS27	12.82 ± 5.01	18.96 ± 3.37	0.001	9.25 ± 2.51	0.009	7.02 ± 4.09	0.001	4.08 ± 5.09	0.000
Active joint count	4.00 ± 1.71	6.08 ± 1.54	0.009	2.00 ± 1.30	0.000	1.54 ± 1.55	0.000	1.00 ± 1.57	0.000
Patient/parent disease activity (VAS 0–10).	4.62 ± 0.84	5.08 ± 0.83	0.047	5.08 ± 0.47	0.027	3.85 ± 1.96	0.014	2.38 ± 2.17	0.000
Physician disease activity (VAS 0–10)	4.38 ± 1.69	6.15 ± 0.77	0.001	3.15 ± 1.35	0.015	2.69 ± 1.20	0.000	1.85 ± 1.41	0.007

(MRI, magnetic resonance imaging; JADAS27, juvenile arthritis disease activity score-27; VAS, visual analogue scale; *P*-values are corrected by Bonferroni test and *P *< 0.013 is considered meaningful).

### 3.4. Medical treatment

In principle, the selection of treatment follows the ILAR guidelines. The medication plan can be timely adjusted according to the examination results to improve the treatment efficacy and promote individualized treatment.

**Figure 3:**
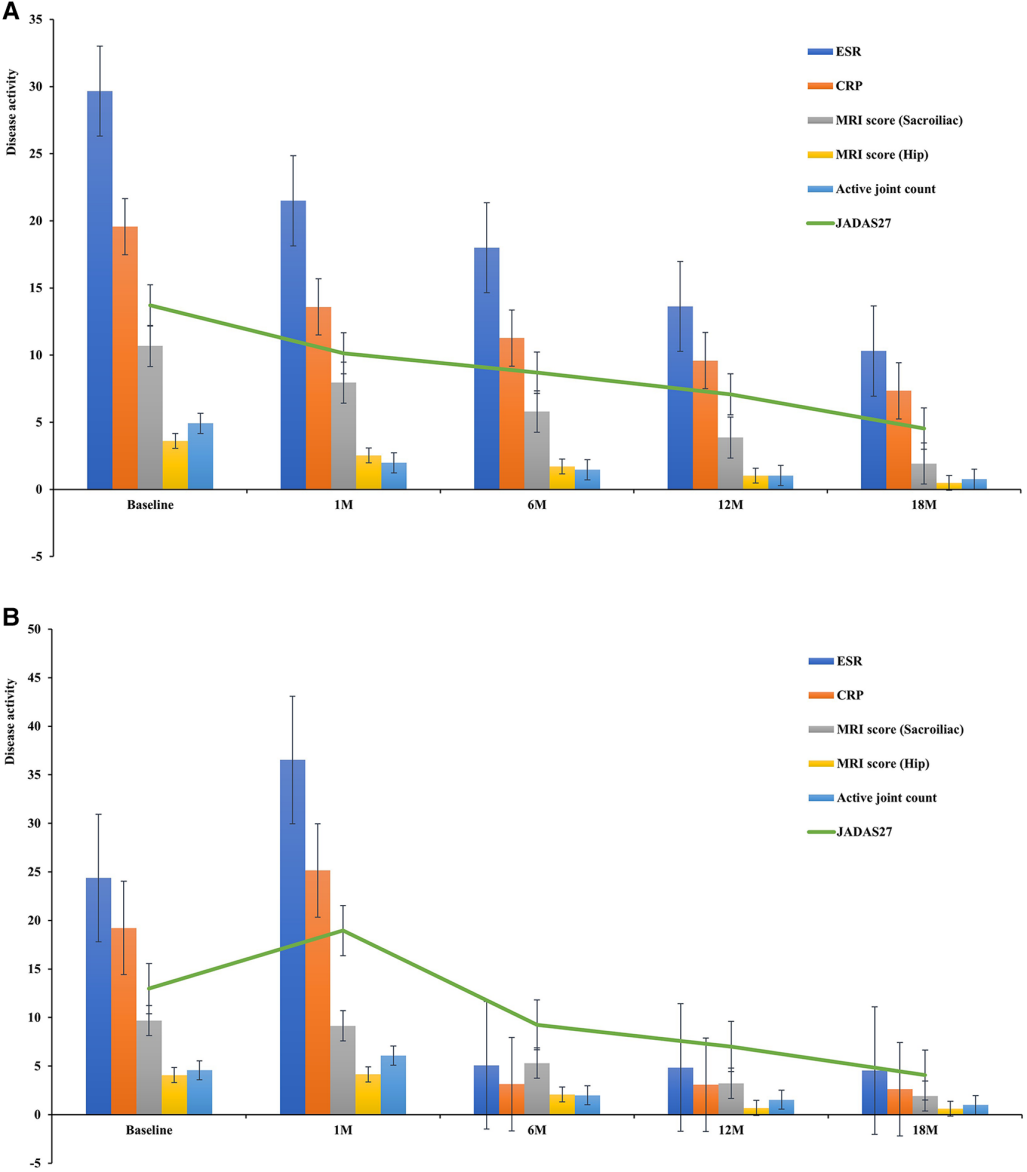
(A) Disease activity after treatment (Group A). (B) Disease activity after treatment (Group B).

In children with ERA who presented to our hospital, after completing the relevant inflammatory indicators (CRP, ESR, TNF-α) and MRI examination, the results showed high inflammatory indicators and poor MRI results; thus, conventional disease-modifying antirheumatic drugs (cDMARDs) combined with biologic disease-modifying antirheumatic drugs (bDMARDs) were usually chosen as the preferred treatment plan. A total of 87 children (64.93%) were treated with biologics, 13 of 87 children with ERA were treated with anti-TNF when they were followed up to 1 month, and the remaining (35.07%) with non-biologics. Of those treated with anti-TNF inhibitors, 71 received etanercept, 13 adalimumab, 2 golimumab, and 1 infliximab. During treatment, 11 children (8.21%) switched to other TNF-α inhibitors. After 1-, 6-, 12-, and 18-month treatment, the clinical symptoms, laboratory indicators, active joint count, JADAS27, and MRI quantitative scores results of Group A improved, without serious adverse reactions (Figure 3A, *P* < 0.013). Thirteen children (9.70%) were administered oral NSAIDs combined with DMARDs at the initial stage of the disease according to disease activity and inflammatory response. However, within one month of follow-up, there were 13 children whose inflammatory markers, MRI quantitative scores, active joint count, JADAS27, CRP, ESR and TNF-α increased or did not improve significantly. Therefore, on the basis of the treatment with cDMARDs, etanercept (12 cases, 92.31%) and golimumab (1 case, 7.69%) were added. After the 18 months follow-up, the disease activity and MRI quantitative scores of the affected children were significantly improved compared with the baseline (Figure 3B, *P* < 0.013). Taking JADAS27 as the comprehensive evaluation criterion, at 12 months of follow-up, a total of 8 (*n* = 74, 10.81%) patients in Group A and 3 (*n* = 13, 23.08%) in Group B reached the state of disease inactivity. At 18 months, a total of 33 patients (*n* = 74, 44.59%) in Group A and 7 (*n* = 13, 53.85%) in Group B reached inactive state. The symptoms and inflammatory index of 9 patients improved significantly after treatment with biological agents. However, MRI showed that BME was relieved one year after treatment with a TNF inhibitor. Symptoms, inflammatory indices, and MRI examinations of three cases treated with cDMARDs and bDMARDs showed no significant improvement from baseline to 18 months.

### 3.5. Security analysis

During the follow-up period, one child treated with a biologic injection exhibited redness, swelling, and itching at the injection site, one presented with tonsillitis and psoriasis, another one experienced streptococcus and mycoplasma infection, and one had latent Mycobacterium tuberculosis infection, these are the concomitant symptoms during the use of etanercept. In addition, there were two cases of liver function damage during the use of etanercept and two cases of adalimumab, which improved after drug withdrawal and the addition of reduced glutathione tablets and hepatoprotective drugs. No other serious adverse events were observed.

## 4. Discussion

This single-center comparative effectiveness study revealed statistically significant improvements associated with active joint counts, JADAS27, CRP, ESR, MRI quantitative scores, and Physician disease activity.

Previous studies have shown that HLA-B27 positivity is a risk factor for the disease to continue into adulthood or transform into adult ankylosing spondylitis, having a good predictive effect on the occurrence and development of the disease; that is, the response to treatment, disease activity, and prognosis of children with HLA-B27 positivity are worse than those with HLA-B27 negativity (17, 18).

ERA usually involves enthesitis, the sacroiliac joints, and the hip, knee, and ankle joints. Extra-articular manifestations are important features of JIA, especially uveitis, which causes visual impairment and blindness in children. An international observational cohort study confirmed differences in the prevalence of JIA-associated uveitis, reporting the highest rates in northern (19.1%) and southern (18.8%) Europe and the lowest in Latin America (6.4%), Africa and the Middle East (5.9%), and Southeast Asia (5.0%) (19). However, only 3.73% (5 patients) of the children in our dataset had uveitis, all with HLA-B27 positivity, and all being negative for antinuclear antibodies. These conclusions bear some limitations, possibly because this study was a single-center retrospective analysis with a small sample size; thus, the results may also be related to different genetic determinants and, perhaps, environmental triggers. These issues should be considered in future genetic analyses, etiopathogenetic investigations, and classification assays. In addition, uveitis is insidious, so the diagnosis is easily missed. Therefore, slit-lamp screening is necessary for children at high risk to minimize the loss of vision or blindness caused by missed diagnoses.

Laboratory tests are essential for assessing affected children's situation and disease activity. Previous research confirmed that CRP, ESR and TNF-α are the main indicators of disease activity in JIA as well as the key evaluation indicators in JADAS27. Our data showed that CRP and ESR decreased from baseline at all stages, from the initial diagnosis to follow-up, with significant differences (*P* < 0.013).

JIA is the most common connective tissue disease among children. ERA is an important subtype of JIA, and the key to treatment is to rapidly relieve pain, control inflammation, and protect children's joint motor function to avoid the decline in quality of life and motor dysfunction caused by untimely treatment or poor effects. However, the current diagnostic criteria lack a clear and objective basis for ERA patients and still rely on clinical manifestations and subjective symptoms. Because joint damage in children is relatively mild, and there are no changes sufficient for the typical imaging diagnosis of x-ray and computed tomography, the latter cannot be used for diagnosis. MRI is considered the most suitable imaging modality for JIA joint lesions in children owing to its unique sensitivity, specificity, and safety. As a sensitive and effective objective examination method, it plays an important role in diagnosing JIA in children, excluding diseases such as infection, trauma, tumor, and muscle strain, and assisting with the classification of JIA (20).

MRI has strong soft tissue discrimination and can clearly show synovitis, bone marrow edema, bone erosion, and other lesions caused by ERA. Severe joint deformities and loss of function may occur in patients with ERA. Early diagnosis, quantitative evaluation, and curative effect observation are areas with the fastest progress in the diagnosis of ERA. MRI is increasingly important role in these aspects and has become the primary imaging method for ERA evaluation.

MRI changes in the inflammation of the sacroiliac and hip joints, including BME, were assessed in patients with ERA using SPARCC and HIMRISS. BME is a sensitive, but relatively nonspecific parameter. Pathological studies have shown that the cause of BME is the infiltration of inflammatory cells into the bone marrow, which reflects the pathological changes in osteitis and is regarded as an early marker of inflammation. This is supported by the evaluation of the activity of related clinical diseases and the concentration of markers (ESR and CRP) in the acute phase (21). Compared to before treatment, the MRI and clinical manifestations of joint inflammation in children improved after TNF-α inhibition treatment (*P* < 0.013). Sometimes, children with ERA do not have symptoms such as joint pain and limitation of motion, but inflammatory reactions in the joints can still be found during MRI re-examination. Therefore, MRI is a key auxiliary examination method for early detection, diagnosis, and treatment.

The current first-line drugs for the treatment of children with ERA are NSAIDs. DMARDs therapy has been widely accepted as a second-line drug for JIA for children with limited mobility, mild bone marrow edema, and fluid accumulation on joint MRI. Methotrexate has good efficacy and safety in the treatment of JIA, and is currently the most widely used DMARD for the treatment of JIA (22). Studies have confirmed that both oral and subcutaneous methotrexate can help improve the condition of children with JIA and ERA, and subcutaneous injection can alleviate gastrointestinal reactions to reduce adverse events in children (23). However, the American College of Rheumatology has recently recommended against using methotrexate monotherapy for children with sacroiliitis (24). Therefore, the treatment plan should differ for each child with ERA. Early suppression of immune and anti-inflammatory treatments is necessary for children with severe diseases.

Recombinant human TNF receptor type II antibody fusion protein is an anti-TNF biological agent; the most commonly used biological agents are etanercept and adalimumab. Several studies have recently confirmed that TNF inhibitors are effective in children with ERA (23), with reductions in inflammatory markers and pain, and better physical function, which is consistent with the current findings. However, due to the small sample size, whether TNF-α inhibitors can reverse joint damage in children remains to be studied.

In recent years, the application of recombinant human type II TNF receptor antibody fusion proteins in children has been widely accepted (25, 26). In 2013, the idea of a “consensus treatment plan”, proposed and formulated by the Childhood Arthritis and Rheumatology Research Alliance, was mainly aimed at the timing of the use of biological agents. There are three schemes in total. The first scheme is the enhancement scheme, whereby treatment biological agents are replaced or added when clinical remission or the expected curative effect has not been achieved after six months of traditional drug treatment. The second and third schemes encompass treatment methods of early combined or separate use of biological agents. Owing to the high price and inconvenience of biological preparations, the first scheme is currently used more frequently (27). The use of biological agents is wise, but their cost is relatively high. Therefore, most children with mild diseases receive DMARDs and NSAIDs in the clinic, mostly with good therapeutic effects. For children with a poor reaction to basic drugs with refractory and severe inflammatory reactions, the addition of TNF inhibitors can significantly alleviate clinical symptoms and inflammatory reactions, while also reversing the MRI joint damage in some children. Another study showed that cDMARDs combined with bDMARDs were significantly better than cDMARDs monotherapy or bDMARDs monotherapy (23). In addition, whether to use bDMARDs in the treatment of children with ERA requires comprehensive clinical symptoms, inflammatory indicators (CRP, ESR, TNF-α, etc.) and MRI examination to evaluate the children's condition, to formulate the best treatment strategy.

Recently, the study of fecal calprotectin (fCAL) levels in children with ERA has gradually entered the relevant research field. The study found that among patients with various subtypes of JIA, the fCAL concentrations were highest in those with the ERA subtype (28), alongside similar studies in adult SpA patients and two studies in children (29, 30). Moreover, the study correlates fCAL concentrations with novel disease activity scores such as JADAS27 and jSpADA, and results show significantly higher fCAL concentrations in ERA patients with active disease (higher inflammatory indicators and MRI signs of sacroiliac arthritis); this suggests that gastrointestinal inflammation may be part of a broader inflammatory process in patients with ERA (28). In addition, because recurrent abdominal pain is common in children, especially those with JIA and/or taking NSAIDs, it is useful to inquire about the underlying cause of inflammation by performing simple, economical, and non-invasive tests such as fCAL (31).

In summary, ERA accounts for a large proportion of patients with JIA in the Asian population. Therefore, it is necessary to formulate effective diagnostic criteria and personalized treatment schemes for children with ERA. Besides, due to the small sample size, there is no statistical analysis on the efficacy of different treatment schemes, so it is necessary to carry out multi center retrospective research. This real-world study is a description and summary of the clinical data of children with ERA in our hospital, so this study not only confirms the effectiveness of tumor necrosis factor inhibitors in children with ERA, but also provides more evidence for precision diagnosis and treatment for other hospitals, families and patients.

## Data Availability

The original contributions presented in the study are included in the article, further inquiries can be directed to the corresponding authors.
